# Bioinformatic analyses identifies novel protein-coding pharmacogenomic markers associated with paclitaxel sensitivity in NCI60 cancer cell lines

**DOI:** 10.1186/1755-8794-4-18

**Published:** 2011-02-11

**Authors:** Lawson Eng, Irada Ibrahim-zada, Hamdi Jarjanazi, Sevtap Savas, Mehran Meschian, Kathleen I Pritchard, Hilmi Ozcelik

**Affiliations:** 1 Fred A. Litwin Centre for Cancer Genetics, Samuel Lunenfeld Research Institute, Mount Sinai Hospital, Toronto, ON, Canada; 2 Department of Laboratory Medicine and Pathobiology, University of Toronto, Toronto, ON, Canada; 3 Department of Pathology and Laboratory Medicine, Mount Sinai Hospital, Toronto, ON, Canada; 4 Sunnybrook Odette Cancer Centre, Sunnybrook Health Sciences Centre, Toronto, ON, Canada; 5 Department of Medicine, University of Toronto, Toronto, ON, Canada

## Abstract

**Background:**

Paclitaxel is a microtubule-stabilizing drug that has been commonly used in treating cancer. Due to genetic heterogeneity within patient populations, therapeutic response rates often vary. Here we used the NCI60 panel to identify SNPs associated with paclitaxel sensitivity. Using the panel's GI50 response data available from Developmental Therapeutics Program, cell lines were categorized as either sensitive or resistant. PLINK software was used to perform a genome-wide association analysis of the cellular response to paclitaxel with the panel's SNP-genotype data on the Affymetrix 125 k SNP array. FastSNP software helped predict each SNP's potential impact on their gene product. mRNA expression differences between sensitive and resistant cell lines was examined using data from BioGPS. Using Haploview software, we investigated for haplotypes that were more strongly associated with the cellular response to paclitaxel. Ingenuity Pathway Analysis software helped us understand how our identified genes may alter the cellular response to paclitaxel.

**Results:**

43 SNPs were found significantly associated (FDR < 0.005) with paclitaxel response, with 10 belonging to protein-coding genes (*CFTR*, *ROBO1*, *PTPRD*, *BTBD12*, *DCT*, *SNTG1*, *SGCD*, *LPHN2*, *GRIK1*, *ZNF607*). SNPs in *GRIK1*, *DCT*, *SGCD *and *CFTR *were predicted to be intronic enhancers, altering gene expression, while SNPs in *ZNF607 *and *BTBD12 *cause conservative missense mutations. mRNA expression analysis supported these findings as *GRIK1*, *DCT*, *SNTG1*, *SGCD *and *CFTR *showed significantly (p < 0.05) increased expression among sensitive cell lines. Haplotypes found in *GRIK1, SGCD, ROBO1, LPHN2*, and *PTPRD *were more strongly associated with response than their individual SNPs.

**Conclusions:**

Our study has taken advantage of available genotypic data and its integration with drug response data obtained from the NCI60 panel. We identified 10 SNPs located within protein-coding genes that were not previously shown to be associated with paclitaxel response. As only five genes showed differential mRNA expression, the remainder would not have been detected solely based on expression data. The identified haplotypes highlight the role of utilizing SNP combinations within genomic loci of interest to improve the risk determination associated with drug response. These genetic variants represent promising biomarkers for predicting paclitaxel response and may play a significant role in the cellular response to paclitaxel.

## Background

Since its approval by the Food and Drug Administration in 1992, paclitaxel (Taxol, Bristol-Myers Squibb, NY) has been commonly used to treat both breast and ovarian cancers. Paclitaxel has also been used in treatment regimens for head and neck cancers, lung cancers, esophageal cancers, testicular cancers and sarcomas [1-4].

Paclitaxel belongs to a family of microtubule-targeting drugs called the taxanes [4]. However, unlike other microtubule-targeting drugs, which cause microtubule instability, paclitaxel stabilizes microtubules leading to the disruption of mitosis (G2/M cell-cycle arrest) and alterations in intracellular communication [2]. Once arrested, cells either remains arrested until the drug is cleared or they begin to undergo apoptosis [4]. The apoptotic mechanism is thought to be independent of microtubule stabilization and is dependent on intrinsic apoptotic pathways leading to caspase activation [5-7]. The JNK, PI3K/AKT and RAF-1 kinase pathways have also been implicated in paclitaxel induced apoptosis [2,4].

Despite aggressive treatment regimens using paclitaxel, response rates are unsatisfactory and vary among groups of patients. The response rates observed from clinical studies of breast cancer patients treated with paclitaxel vary from 21-86% [2]. Similarly in ovarian cancer, the response rate varied from 20-65% and in non-small lung cancer, 30-56% [2]. One approach to improve drug response is by increasing dosages beyond the typical dose to increase the efficacy, but this is not an available approach due to the existence of dose limiting toxicities. In the case of paclitaxel, these toxicities include myelotoxicity, neurotoxicity and neutropenia [2].

Although the observed variation can be accounted for by differences in environmental factors such as age or treatment compliance, it may also be attributed to differences among the genetic profiles of patients [8]. These genetic variations are found to impact the activities of genes involved in the drug's pharmacokinetics, its cellular targets or other signalling pathway proteins downstream of the target [8]. These genetic variations include single nucleotide polymorphisms (SNPs), micro-satellites or copy number variation [8].

Here, we used a methodology previously developed by our group for studies on gemcitabine and selenium, and expanded upon it to identify genetic variants associated with the cellular response to paclitaxel [9,10]. We used the NCI60 cancer cell line panel, a collection of genetically well-characterized patient tumours to help us identify these variants. This panel has been commonly used to screen numerous therapeutic agents, including paclitaxel [11]. We integrated the paclitaxel screening results for the NCI60 panel with the panel's SNP data to perform a genome-wide association study (GWAS). Bioinformatic tools were applied to understand how these genetic variants alter paclitaxel response. Lastly, we performed a haplotype analysis on the protein-coding genes containing potential markers to identify haplotypes that were more strongly and significantly associated with paclitaxel response than the single SNP markers alone. In turn, we feel that these variants and haplotypes will help to serve as potential biomarkers, identifying patients that will best respond to paclitaxel.

## Methods

### Case-control design of the NCI60 drug response data

The cellular response data for the NCI60 panel to paclitaxel treatment was acquired through Developmental Therapeutics Program (DTP), a branch of the National Cancer Institute (http://dtp.nci.nih.gov/). Specifically, we obtained the log 10 value of the growth inhibitory 50% (GI50) response data (effective concentration that arrests cellular growth in 50% of a population of cells) for the NCI60 panel. The GI50 response data was determined from a series of measurements taken from a starting dose of 10^-6 ^M. Specific information about the determination of GI50 values can be found on the DTP website (http://dtp.nci.nih.gov/branches/btb/ivclsp.html). A total of 62 cell lines had drug response data available from this drug screening protocol. To help categorize specific cell lines as either sensitive or resistant, we followed a methodology previously used by our group using the statistical software package, SAS 9.1 (SAS Institute, Cary, NC) [9]. On all 62 cell lines, we converted the response data to normalized z-scores and then performed a non-parametric kernel density estimation on these z-scores to help with the categorization.

### Statistical analysis of genome wide genetic data

In order to identify SNPs that are significantly associated with drug response, we obtained the Affymetrix 125 k SNP array (Affymetrix, Santa Clara, CA) genotype data (http://dtp.nci.nih.gov/mtargets/download.html) for the NCI60 panel. A total of over 124,000 SNPs were genotyped on this array, but the data for only 118,409 SNPs was available [12]. Drug response data for the cell lines from the DTP website were matched with the genomic data available on this SNP array. Only cell lines which had genomic data available from this array were used in the GWAS. This reduced the number of cell lines analyzed from 62 to 58. A GWAS of the drug response to the SNP-genotype data was carried out using whole genome association analysis software, PLINK (http://pngu.mgh.harvard.edu/~purcell/plink/) [13]. Cell lines missing more than 25% of their genotype data or SNPs with a call rate lower than 75%, or a minor allele frequency of less than 2%, were excluded from the study. As a result of these quality control measures, 20,514 SNPs were removed for missing more than 25% of the genotype data, 20,176 SNPs were removed for having a minor allele frequency of less than 2%, leaving 79,622 SNPs in the GWAS. SNPs were considered significantly associated with drug response if they had a false discovery rate (FDR) q value (following Benjamini and Hochberg (1995)) of less than 0.005. These significant SNPs were mapped back to their corresponding genes based on information provided by dbSNP Build 131 (http://www.ncbi.nlm.nih.gov/projects/SNP/). Information about the description of each gene was obtained from the online database, GeneCard (http://www.genecards.org).

### Impact of significant variants on respective genes

Functional prediction was performed for each SNP identified to determine its potential effect on its gene product. This was conducted using the online software, FastSNP (http://fastsnp.ibms.sinica.edu.tw/) [14]. This software follows a specific algorithm in order to determine the possible functional effect of the variant on the gene (i.e., intronic enhancer, missense mutation) and the associated risk of having the specific SNP variant. FastSNP utilizes information available from another online program PolyPhen (http://www.bork.embl-heidelberg.de/PolyPhen/) to predict whether any SNPs causing missense mutations will also cause a non-conservative change in protein structure [15]. In addition, FastSNP uses information available from three different web resources, namely ESEfinder, Rescue-ESE and FAS-ESS to determine whether SNPs are located at exonic splicing regulatory sites and if they will cause changes in splicing regulation [16-18].

### Interactome analysis and impact of genes on drug response

To learn how the identified genes interact with each other and other cellular proteins and also to understand their role in the cellular response to paclitaxel, pathway-based analysis was conducted using Ingenuity Pathway Analysis software (Ingenuity Systems Inc, Redwood City, CA) as of April 2010. Specifically, direct protein-protein interaction, gene regulation information and gene expression change information was used in this investigation. Furthermore, we conducted our own literature investigation on each protein-coding gene using PubMed to understand how each gene may affect paclitaxel response.

### mRNA expression analysis

mRNA expression data for the NCI60 panel, as measured on the Affymetrix U133A Chip and normalized using the per-gene normalization to the median approach, was obtained from the online database, BioGPS (http://biogps.gnf.org) [19]. Protein-coding genes which lacked available probes were excluded from this analysis. As well, cell lines which did not have corresponding mRNA expression data available were also excluded from the analysis. For genes with multiple probes, each probe was used as an independent measure of the expression level. In some cases, where cell lines had two sets of expression measurements (HCT116, HL60, NCIH23A), we averaged the two sets and treated the averaged expression as a single set of measurements for the cell line. Case-control analysis was conducted for each probe between the mRNA expression level in the sensitive group and the levels in the resistant group. A Mann-Whitney U-test was used as a measure of significance with p values less than 0.05 being considered statistically significant. Due to the exploratory nature of this study, we did not perform corrections for multiple comparisons.

### Haplotype analysis

In order to test whether multiple SNPs from the same gene region can more significantly (with respect to unadjusted p values) and strongly predict drug response, we performed haplotype association analysis based on the additional SNP data flanking the significantly associated SNPs within the identified protein-coding genes. This was performed using the software, Haploview (http://www.broad.mit.edu/haploview/haploview) [20]. For each protein-coding gene identified, we inputted into Haploview, the remaining SNPs belonging to the same gene and their associated genotype data from the Affymetrix 125 k SNP array. Cell lines that were missing 50% of their data were excluded from this analysis. As well, SNPs missing 75% of their genotype data or had a minor allele frequency less than 0.1% were also excluded. The odds ratio and 95% confidence interval of the odds ratio for each haplotype was calculated to determine the confidence of the statistical results obtained. Since this haplotype investigation was also exploratory in nature, we chose to not perform corrections for multiple comparisons.

## Results

### Case-control Study

**Figure 1:**
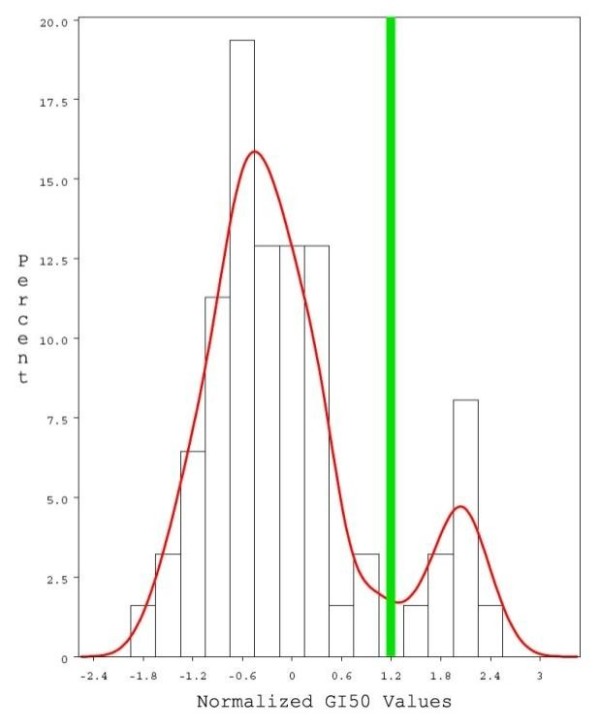
**Categorization of cell lines in the NCI60 panel into paclitaxel sensitive and resistant groups**. This graph was generated by SAS 9.1 and shows the distribution of the panel's response data to paclitaxel after a non-parametric kernel estimation with bandwidth of 0.55 was applied. The 62 cell lines which had drug response data available from DTP were used in this categorization. Normalized drug response z-scores are listed on the x-axis and relative frequency in each bin on the y-axis. The vertical line indicates the location of the antimode in the distribution and the z-score value (1.2) which determined whether cell lines were categorized as sensitive or resistant.

The non-parametric kernel density estimation of the NCI60 GI50 response data helped to identify the optimum cut-off z-score, thus categorizing the cell lines into a resistant group (n = 8) if their z-scores were greater than 1.2 (10^-6.807 ^M) and a sensitive group (n = 50) if they had z-scores less than 1.2. The distribution of our drug response data and the kernel estimation applied, which had a bandwidth of 0.55 can be found in Figure 1 with specific cell line listings in Additional File 1, together with the information on whether cell lines had SNP genotype data available.

Our GWAS analysis revealed 43 SNPs that were significantly associated with paclitaxel response (FDR q value < 0.005). A listing of these SNPs, brief descriptions of the identified genes along with additional statistics (unadjusted p-values, chi-squared and Bonferroni correction) can be found in Table 1. Ten of these SNPs were found to be located within the protein-coding genes; in the order of significance [Syntrophin, gamma 1 (*SNTG1*) (q = 6.78E-05); Glutamate receptor, ionotropic, kainate 1 (*GRIK1*) (q = 0.00125); Dopachrome tautomerase (*DCT*) (q = 0.00208); BTB (POZ) domain containing 12 (*BTBD12*) (q = 0.00248); Sarcoglycan, delta (*SGCD*) (q = 0.00319); Roundabout, axon guidance receptor, homolog 1 (*ROBO1*) (q = 0.00335); Protein tyrosine phosphatase, receptor type, D (*PTPRD*) (q = 0.00354); Cystic fibrosis transmembrane conductance regulator (*CFTR*) (q = 0.00416); Zinc finger protein 607 (*ZNF607*) (q = 0.00423); Latrophilin 2 (*LPHN2*) (q = 0.00477)]. For the SNP markers whose frequency was zero in either the sensitive or resistant group, the odds ratio (OR) and 95% confidence interval for the odds ratio were unavailable from PLINK.

**Table 1:** Significant SNPs (FDR q value < 0.005) associated with paclitaxel response along with statistical information

CHR	Marker	RS_ID	Gene Name	Gene Description	A1	AF_R	AF_S	A2	UNADJ	BONF	FDR_BH	CHISQ	OR	L95	U95
1p31.1	91699	rs371363	LPHN2	Latrophilin 2, G-protein coupled receptor	A	0.5	0.0714	G	2.58E-06	0.2051	0.00477	22.11	13	3.741	45.17

2q14	1413956	rs1898705			A	0.571	0.0851	G	1.76E-06	0.1405	0.003902	22.84	14.33	3.975	51.69

2q14.3	1421343				C	0.438	0.0313	A	1.32E-07	0.01052	0.001169	27.84	24.11	5.296	109.8

2q31.2	1481588				T	0.25	0	C	1.02E-06	0.08144	0.003192	23.88	NA	NA	NA

2q33.1	1503580				A	0.5	0.0375	G	1.26E-06	0.1006	0.003354	23.48	25.67	4.721	139.5

2q35	1523870	rs6739040			A	0.286	0	G	1.39E-06	0.1104	0.003451	23.3	NA	NA	NA

2q36.3	1538316				T	0.375	0.0213	C	4.74E-07	0.03773	0.002331	25.37	27.6	4.902	155.4

3p24.3	1743877				C	0.286	0	A	2.32E-07	0.0185	0.001542	26.74	NA	NA	NA

3p14.2	1785132				G	0.333	0.0106	C	6.86E-07	0.05459	0.002481	24.66	46.5	4.628	467.2

3p12.3	1805457	rs1032966	ROBO1	roundabout, axon guidance receptor, homolog 1 (Drosophila)	A	0.333	0.0111	G	1.20E-06	0.09528	0.003354	23.58	44.5	4.428	447.3

3q26.1	1876142	rs2404571			G	0.667	0.0854	A	2.81E-07	0.02238	0.001721	26.38	21.43	5.136	89.41

4p15.3	1929743	rs685064			C	0.313	0.0114	T	2.01E-06	0.1602	0.004162	22.58	39.55	4.224	370.3

5q23.2	2254477	rs959300			G	0.714	0.1111	A	9.98E-08	0.007946	0.001135	28.38	20	5.274	75.84

5q33.3	2292501	rs7715464	SGCD	sarcoglycan, delta (35kDa dystrophin-associated glycoprotein)	A	0.563	0.0851	G	1.04E-06	0.08298	0.003192	23.85	13.82	4.06	47.05

5q33.3	2294131				A	0.313	0.0102	G	5.15E-07	0.04098	0.002331	25.21	44.09	4.714	412.4

6p25.1	2325528	rs2073042			A	0.786	0.1622	G	1.12E-06	0.08914	0.003301	23.71	18.94	4.587	78.25

6p22.3	2343860				A	0.917	0.141	C	5.87E-09	0.000467	0.000156	33.88	67	7.85	571.8

6q14	2403189				A	0.333	0.0116	G	2.09E-06	0.1665	0.004162	22.51	42.5	4.227	427.3

6q22.1	2440993	rs594930			C	0.4	0.0217	T	1.38E-06	0.1098	0.003451	23.31	30	4.542	198.2

7q31.2	2626491	rs213988	CFTR	Cystic fibrosis transmembrane conductance regulator	G	0.438	0.0444	A	2.03E-06	0.1616	0.004162	22.57	16.72	4.093	68.31

8p12	2715222				G	0.25	0	A	1.72E-06	0.1373	0.003902	22.88	NA	NA	NA

8q11.22	2727769	rs2385525			C	0.429	0.0217	T	7.93E-08	0.006315	0.001052	28.82	33.75	5.829	195.4

8q11.22	2727770	rs2132528			G	0.5	0.0256	A	3.77E-08	0.003005	0.00063	30.26	38	6.591	219.1

8q11.22	2727954				T	0.583	0.0778	G	1.75E-06	0.1392	0.003902	22.85	16.6	4.165	66.17

8q11.22	2728154	rs318885	SNTG1	syntrophin, gamma 1	G	0.6	0.025	T	1.70E-09	0.000136	6.78E-05	36.29	58.5	8.841	387.1

8q24.21	2806750	rs2568409			G	0.643	0.0455	T	4.79E-10	3.81E-05	3.81E-05	38.76	37.8	8.573	166.7

9p23	2835786				G	0.25	0	A	6.07E-07	0.04836	0.002418	24.89	NA	NA	NA

9p23	2836025	rs7470838	PTPRD	protein tyrosine phosphatase, receptor type, D	T	0.857	0.2245	C	1.47E-06	0.1168	0.003538	23.19	20.73	4.311	99.66

9p23	2836880				T	0.714	0.1413	C	1.26E-06	0.1005	0.003354	23.48	15.19	4.143	55.72

9p22.2	2845463				G	0.25	0	A	7.88E-07	0.06275	0.002728	24.39	NA	NA	NA

9p21.2	2856599	rs2060439			G	0.25	0	A	6.07E-07	0.04836	0.002418	24.89	NA	NA	NA

9q21.33	2897567				G	0.6	0.0488	A	1.24E-07	0.009894	0.001169	27.95	29.25	5.814	147.2

11q14.1	494182	rs7927911			A	0.429	0.0256	C	8.37E-07	0.06661	0.002775	24.27	28.5	4.912	165.4

12q21	629187				G	0.25	0	A	3.61E-07	0.02874	0.002053	25.89	NA	NA	NA

13q13.3	709113	rs7335400			A	0.563	0.0714	C	1.58E-07	0.01257	0.001251	27.49	16.71	4.781	58.43

13q21.33	748668				G	0.857	0.1628	A	3.96E-08	0.003151	0.00063	30.17	30.86	6.213	153.2

13q32.1	779846	rs727299	DCT	dopachrome tautomerase	T	0.313	0.01	C	3.92E-07	0.03122	0.002081	25.73	45	4.812	420.8

16p13.3	997458	rs714181	BTBD12	BTB (POZ) domain containing 12	A	0.313	0.0104	G	6.76E-07	0.05379	0.002481	24.68	43.18	4.616	404

16p13.12	1011445	rs251919			C	0.438	0.0385	T	2.41E-06	0.1918	0.004566	22.24	19.44	4.257	88.81

16q23.3	1071765				G	0.571	0.0745	C	5.27E-07	0.04196	0.002331	25.16	16.57	4.476	61.35

18p11.22	1162169	rs3975417			T	0.333	0.0116	C	2.09E-06	0.1665	0.004162	22.51	42.5	4.227	427.3

19q13.12	1266548	rs958305	ZNF607	zinc finger protein 607	C	0.5	0.061	T	2.18E-06	0.1738	0.004239	22.43	15.4	4.058	58.44

21q21.3	1647581	rs457531	GRIK1	glutamate receptor, ionotropic, kainate 1	T	0.5	0.0349	C	1.73E-07	0.01377	0.001251	27.31	27.67	5.504	139.1

Significant SNPs were traced back to their genes using dbSNP Build 131 in order to identify which protein-coding genes they belonged to. (CHR = Chromosome loci, Marker = SNP ID number on Affymetrix 125 k SNP Array, RS_ID = SNP rs number, Gene Name = Notation for gene as found on Human Genome Organization Gene Nomenclature Committee website, A1 = Associated SNP allele, A2 = Alternate variant allele, AF_R = Frequency of associated allele in resistant cell lines, AF_S = frequency of associated allele in sensitive cell lines, UNADJ = unadjusted p value, BONF = Bonferroni correction value, FDR_BH = FDR correction value, CHISQ = Chi-Squared Value, OR = Odds Ratio, L95 = Lower Bound of 95% confidence interval for odds ratio, U95 = Upper Bound of 95% confidence interval for odds ratio). Statistics with NA written indicate that the statistic could not be calculated. This GWAS was performed using only 58 of the 62 cell lines which had drug response data available since the remaining 4 cell lines lacked available genotype data.

### Impact of significant variants on respective genes

Among the ten significant SNPs mapped to protein-coding genes, eight could be analyzed using FastSNP [14], while no FastSNP data was available on the remaining two (*PTPRD, SNTG1*). Four of these eight SNPs were located within the intronic sequences acting as potential intronic enhancers *(GRIK1*, *DCT*, *SGCD*, and *CFTR*). SNPs belonging to *ROBO1 *and *LPHN2 *were also located within intronic sequences, but were not predicted to have a functional consequence on gene expression and/or function. The variants in *ZNF607 *and *BTBD12 *were found to cause conservative missense mutations (*ZNF607*-R531K, *BTBD12*-P1122L) with predicted benign effects on protein structure/function Additionally, the variant in *ZNF607 *was predicted to cause changes in splicing regulatory effects. Specifically for the two variants (C/T) of *ZNF607 *in this investigation, cell lines having the C variant were predicted to not have exonic splicing silencer (ESS) sites, while those with the T variant were predicted to have three ESS sites. Investigation of the SNPs in *SNTG1 *and *PTPRD *using GeneView on dbSNP revealed that they lied within the intronic regions of their respective genes. However, their potential effects on gene expression or protein function remain unknown.

### Interactome analysis and impact of genes on drug response

Using Ingenuity Pathway Analysis and a complementary PubMed literature search, we have investigated the biological interactions amongst the novel candidate markers and how they may alter the cellular response to paclitaxel. This analysis identified additional key proteins (such as p53, β-catenin (CTNNB1), ERBB2) commonly involved in cancer pathways. These investigations helped us classify the identified genes into two groups when determining the cellular response to paclitaxel: those such as *PTPRD *and *BTBD12*, interacting with the p53 and β-catenin axis, and those including *ROBO1, CFTR, ZNF607, GRIK1, LPHN2, DCT, SGCD, SNTG1 *that interact with cellular microtubules.

### Gene expression analysis

mRNA expression data available on the Affymetrix U133A chip was subjected to case-control analysis using the Mann-Whitney U Test (Table 2). Only 54 NCI60 cell lines had mRNA expression data available (Additional File 1). Individual probes were available to investigate the expression of all genes, but *BTBD12 *and *ZNF607*, which did not have any probes. The mRNA expression analysis revealed significantly different expression levels between sensitive and resistant cell lines for a total of 15 probes belonging to five genes (*DCT*, *SNTG1*, *CFTR*, *GRIK1*, and *SGCD*). In all cases where there was a significant difference in expression level, sensitive cell lines always showed greater expression levels when compared to resistant cell lines.

**Table 2:** mRNA expression case-control analysis results for the NCI60 panel using mRNA expression data from BioGPS

Gene	Probes	p value	Gene	Probes	p value
LPHN2	206953_s_at	0.6612		205337_at	0.0062
		
ROBO1	213194_at	0.5188		205338_s_at	0.0098
			DCT		
	213543_at	0.0095		216512_s_at	0.0053
			
	214492_at	0.0026		216513_at	0.0049
SGCD					
	210329_s_at	0.0031		214611_at	0.0077
			GRIK1		
	210330_at	0.0053		207242_s_at	0.0243

	215702_s_at	0.0293		205712_at	0.7332
			
	215703_at	0.0095	PTPRD	213362_at	0.6878
CFTR					
	217026_at	0.0117		214043_at	0.7795
	
	205043_at	0.0074	SNTG1	220405_at	0.0154

Protein-coding genes which did not have any available probes to measure their expression (*BTBD12*, *ZNF607*) were excluded from this analysis along with cell lines that did not have data available. A total of 20 probes were available to help analyze the expression pattern of the 8 genes listed above. As well, only 54 of the cell lines (from the 58 used in the GWAS) were used in this analysis as the remainder lacked gene expression data. Expression differences between sensitive and resistant cell lines were considered to be significant if p < 0.05 for the Mann-Whitney U-test.

### Haplotype analysis

**Figure 2:**
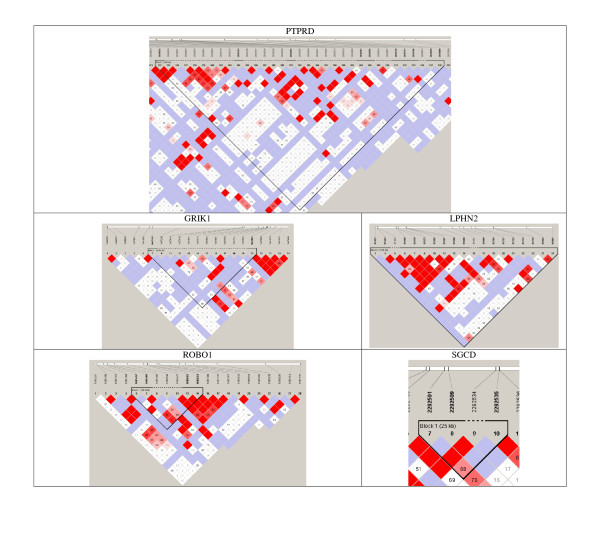
**Results of haplotype analysis using Haploview software**. Haplotype analysis was performed to identify haplotypes more significantly and strongly associated with drug response than the originally identified SNP. Solid lines represent SNPs that were used in the haplotype analysis and are part of the haplotype from SNP block whereas dashed lines represent SNPs that were used in the analysis, but were not part of the haplotype. The specific nucleotides, frequencies and significance values can be found in Table 3.

Haplotype analysis was performed using Haploview to identify haplotypes within our list of protein-coding genes that had stronger association with drug response than the individual SNP markers alone. Each of these haplotypes contained the SNP that was detected as being associated with the cellular response to paclitaxel from our GWAS analysis. Five protein-coding genes [*LPHN2 *(TTGAGCATCATCTCCCC, p_SNP _= 2.58E-06 vs. p_haplotype _= 2.71E-08), *PTPRD *(TGGATCCCGT, p_SNP _= 1.47E-06 vs. p_haplotype _= 4.62E-10), *GRIK1 *(GT, p_SNP _= 1.73E-07 vs. p_haplotype _= 1.01E-07), *ROBO1 *(AGGT, p_SNP _= 1.2E-06 vs. p_haplotype _= 7.91E-08), and *SGCD *(GAC, p_SNP _= 1.04E-06 vs. p_haplotype _= 4.12E-07)] had haplotypes that were more significantly associated with drug response. Additionally, these five haplotypes were also more strongly associated with drug response based on their odds ratio [*GRIK1 *(OR_SNP _= 27.67 vs. OR_Haplotype _= 30.28), *ROBO1 *(OR_SNP _= 44.50 vs. OR_Haplotype _= 84.25), *SGCD *(OR_SNP _= 13.82 vs. OR_Haplotype _= 36.70), *PTPRD *(OR_SNP _= 20.73 vs. OR_Haplotype _= 38.85), *LPHN2 *(OR_SNP _= 13.00 vs. OR_Haplotype _= 23.73]. These haplotypes can be found in Table 3. A figurative illustration of the relative positions of the SNPs in their protein-coding genes is shown in Figure 2.

**Table 3:** Summary of case-control analysis of haplotypes associated with paclitaxel response

Gene	Haplotype	Haplotype Frequencies	Case, Control Ratio Counts	Case, Control Frequencies	Chi Square	p Value	Odds Ratio	L95	U95
***GRIK1***	GT	0.073	6.3 : 9.7, 2.1 : 97.9	0.394, 0.021	28.362	1.01E-07	30.28	5.56	164.98

***ROBO1***	AGGT	0.043	4.1 : 7.9, 0.6 : 97.4	0.339, 0.006	28.828	7.91E-08	84.25	5.10	1391.73

***SGCD***	AAC	0.043	4.0 : 12.0, 0.9 : 99.1	0.251, 0.009	19.847	8.39E-06	36.70	3.45	390.22

***PTPRD***	TGGATCCCGT	0.095	8.3 : 7.7, 2.7 : 97.3	0.518, 0.027	38.832	4.62E-10	38.85	8.19	184.29

***LPHN2***	TTGAGCATCATCTCCCC	0.12	8.0 : 6.0, 5.0 : 89.0	0.571, 0.053	30.902	2.71E-08	23.73	5.91	95.28

This table summarizes the haplotypes that were found more significantly associated with drug response than the originally identified SNP marker alone. Cell lines categorized as cases here are those which were in the resistant group and those categorized as controls are those in the sensitive group. Figure 2 illustrates the relative position of these SNP markers along the genes. Statistics with NA written indicate that the statistic could not be calculated.

## Discussion

### Novel method development and application

In this study, we have built upon a previous methodology using the NCI60 panel to identify genetic markers associated with drug response: namely gemcitabine and selenium by our group and perifosine by Zhang *et al *[9,10,21]. Instead of investigating specific candidate biological processes/pathways, here we used a genome wide approach with an aim of identifying genomic variants not originally thought to be related to the drug's mechanism. Previous studies on paclitaxel have investigated the pharmacogenetics of specific genes associated with paclitaxel response, genes associated with paclitaxel metabolism, and cDNA, protein activation status and siRNA changes associated with response [22-26]. However, this is the first genome-wide SNP association study done for paclitaxel response. Furthermore, the use of cell lines in these types of GWAS studies allowed access to high throughput genomic and transcriptomic data, along with robust drug screening protocols allowing for more accurate results and also allowed for greater control of non-genetic factors such as treatment compliance and administration time [27,28].

### Relevance to paclitaxel dosing in clinical settings

The GI50 response data was extrapolated from a range of doses which started at a 10^-6 ^M dose and this was used as the basis for our case-control analysis. Although this dose was above therapeutic concentrations, the actual drug concentrations determined to cause 50% growth inhibition (Additional File 1) ranged from 0.104 nM to 7.96 μM. The minimum is below the therapeutic concentration range's minimum of 100 nM, while our maximum is slightly below the maximum therapeutic concentration 10 μM [29]. This supports the clinical relevance of the GI50 values utilized in this study.

### Impact of significant variants on respective genes and gene expression

Using FastSNP, four of the SNPs were found as intronic enhancers of their respective genes (*SGCD*, *DCT*, *GRIK1*, and *CFTR*). This suggests that the SNP variant does not alter the protein product significantly, but may alter transcription factor binding leading to altered expression patterns [14]. In all cases, these genes were found to have significantly increased mRNA expression in sensitive cell lines for all probes available, supporting their predicted effect by FastSNP. The differential mRNA expression shown for *SNTG1 *suggests that its intronic variant may also serve as an intronic enhancer. Since only half of our protein-coding genes showed significant differences in mRNA expression, this emphasizes the importance to investigate genetic changes as the remaining five genes (*ROBO1*, *LPHN2*, *PTPRD*, *BTBD12 *and *ZNF607*) would not have been detected solely based on mRNA expression data. As well, these suggests that mechanisms other than changes in that specific gene's expression may help to explain how our identified SNPs can alter paclitaxel response. For the remaining protein-coding genes with intronic SNP variants: *ROBO1*, *LPHN2 *and *PTPRD*, it remains unclear how their variant may alter their gene product. However, SNPs located in intronic and intergenic regions can regulate the expression of many other genes (located on the same chromosome or different chromosomes); serving as master regulators and this may be how our intergenic and remaining intronic variants can be altering paclitaxel response [30]. In the case of *BTBD12 *and *ZNF607*, their variants each caused conservative missense mutations with predicted benign effects on protein structure. These variants would not cause major changes in protein or cellular function but may still be significant enough to alter paclitaxel response. As well, the potential alternative splicing of *ZNF607 *can lead to further changes in the protein product, altering drug response. Alternatively, these SNPs may be in linkage disequilibrium with the real genetic factor that has a biological consequence directly or indirectly contributing to the paclitaxel response. Although FastSNP did accurately predict the potential effect for the 4 SNPs serving as intronic enhancers along with the intronic SNPs in *ROBO1 *and *LPHN2 *as not having any effects, these predicted changes are only suggestive at this stage and will need further validation using; for example, *in vitro *cell based functional assay systems. In addition, since the mRNA expression data from BioGPS served as adjunctive support for our findings, this data will need further validation by Real-time PCR or Northern blot analysis.

### Haplotype blocks associated with paclitaxel response

In addition to individual SNP markers, we searched for haplotypes that were more significantly and strongly associated with paclitaxel response and hence can be more informative as response predictors. A total of five protein-coding genes had such haplotypes. In each of these haplotypes, our original SNP marker was found as part of the haplotype. Many of the remaining SNPs in the haplotypes were not found significantly associated with paclitaxel response when working alone and would have remained undetected unless they were working in concert with the original SNP marker. This supports the synergistic effect that multiple SNPs can exert on a gene which has been found to occur in processes such as cancer metastasis [31]. This synergism can occur among SNPs in the same gene or between different genes [32-34]. The location of the SNPs in these haplotypes may also help to identify different gene regions or functional domains that can directly impact drug response and provide more insight into the drug's mechanism. Furthermore, one of our identified SNPs at 9p23 (marker number: 2836880) was found as part of the identified *PTPRD *haplotype. This suggests that by performing haplotype analyses, SNPs identified in the GWAS that were originally unclear on their role in paclitaxel sensitivity, may be found related to part of the gene through haplotypes.

### Impact of genes on drug response

Our GWAS identified 10 genes containing SNP variants associated with paclitaxel response. Due to the *in vitro *nature of our study, it identifies only genes playing a direct role in the cellular response to paclitaxel, but do not reveal any variation potentially associated with paclitaxel's pharmacokinetics. Hence, the SNPs such as those previously identified in the drug metabolizing enzymes: *CYP2C8*, *3A4/5 *and *2C19 *and drug transport proteins: p-glycoprotein (*MDR1*) and *SLCO1B3 *would not be identified here [3,22,25]. Nevertheless, our analysis suggested that the genes identified here fall into two groups: those altering paclitaxel response through the β-catenin and p53 transcriptional regulatory axis, and those altering paclitaxel response through microtubule interactions.

#### 1. β-catenin and p53 axis and paclitaxel response

Mutations of p53 and β-catenin have commonly been found associated with cancer risk and progression and in the case of p53, also response to chemotherapy [35,36]. β-catenin plays a role in cell growth/morphogenesis, cell polarity and cell adhesion and induces expression of p53 and related candidates, which in turn regulates β-catenin's transcriptional activity [37-39]. p53 detects DNA damage and its activation can lead to cell cycle arrest and/or apoptosis [38,39]. p53 is directly associated with paclitaxel chemosensitivity and paclitaxel increases p53 expression, phosphorylation status and its nuclear accumulation leading to the arrest of cell growth and apoptosis [4,40-42].

Two of our identified genes *PTPRD *and *BTBD12 *interact with these two genes or their related pathways and may impact paclitaxel response through them. p53 decreases expression of protein tyrosine phosphatase receptor type A (PTPRA) which interacts with our candidate, PTPRD [43,44]. PTPRA and PTPRD are receptor tyrosine phosphatases, and PTPRD can dephosphorylate v-src sarcoma viral oncogene homolog (SRC), altering v-raf-1 murine leukemia viral oncogene homolog 1 (RAF-1) activation, a major pathway for paclitaxel induced apoptosis [2,6,45,46]. BTBD12 is a scaffold protein that helps to regulate various DNA repair enzymes including those used for double stranded breaks and cross-links and also interacts with enzymes for cell-cycle control, homologous recombination and replication forks [47-50]. In addition to arresting cell division, paclitaxel can also induce DNA damage, while repressing various DNA repair genes leading to cytotoxicity [51]. Thus, the missense substitution caused by *BTBD12*'s variant could reduce its DNA repair ability leading to increased paclitaxel susceptibility or alter paclitaxel's ability to arrest mitosis.

#### 2. Microtubule interactions and paclitaxel response

Microtubules play an important role in paclitaxel response. Paclitaxel binds to β-tubulin when it is present as assembled tubulin and causes cell-cycle arrest, and eventually apoptosis [4]. Previous studies have found that point mutations in tubulin, which weaken drug-tubulin interactions and altered expression of a microtubule stabilizing protein, microtubule-associated protein 4 (*MAP4*), both alter paclitaxel response in cells [2,4].

Several of our identified genes may also affect paclitaxel response through changes in microtubules dynamics or stability. ROBO1 is a neuronal receptor used in neurodevelopment and when activated by its ligand Slit, directly interacts with the beta 4 tubulin subunit (TUBB4) of microtubules to regulate microtubule dynamics [52-55]. CFTR is a chloride transporter channel and is required for proper microtubule distribution, as studies have shown that cells having mutant CFTR (DeltaF508) show microtubular disorganization [56]. ZNF607 is a zinc finger protein involved in transcriptional regulation and it interacts with Ataxin 1 (ATXN1), which interacts with microtubules to alter cell morphology during neuronal development [54,57,58].

As well, many of our identified genes use microtubules for their own transport or transport of other proteins and therefore these genes can also cause changes in microtubule dynamics, potentially altering paclitaxel response. GRIK1 is a kainite receptor used for interneuronal communication and uses microtubules to get transported to the surface of dendrites [59,60]. Additionally, GRIK1 can indirectly affect cellular binding to paclitaxel through co-regulation of expression by Atrophin 1 (ATN1), which also regulates Stathmin 1 (*STMN1*) expression and STMN1 reduces cellular binding of paclitaxel [61-63]. LPHN2 interacts with microtubules when it binds to a ligand, α-Latrotoxin in order to cause neurotransmitter release [64]. DCT belongs to a group of enzymes used in the biosynthesis of melanin and this process uses microtubules to transport melanosomes containing melanin, to the cell surface [65,66]. However, *DCT *is also a marker for melanoma and the observed results for this gene may be caused by the melanoma cell lines all belonging to the sensitive cell line category [67].

The last two of our genes, *SGCD *and *SNTG1 *which represent members of their respective families, sarcoglycans and syntrophins form a part of a larger complex called the dystrophin associated glycoprotein complex (DAGC). The DAGC connects the cytoskeleton to the extracellular matrix in muscle [68]. The beta isoform of sarcoglycan, SGCB along with SGCD have been found to co-localize with tubulin during the M phase of the cell cycle, and this corresponds to the cell cycle stage when paclitaxel interacts with tubulin [4,25,69]. Members of the syntrophin family, including β2-syntrophin have been found to associate with purified microtubules through a PDZ domain which is shared by SNTG1 [70,71]. The association of these genes with microtubules suggests that they may play a role in regulating microtubule dynamics; thereby potentially altering paclitaxel response.

The NCI60 panel comprises of a limited number of human cancer-derived cell lines, and as such, there may be confounding by either race or tumor type in our identification of pharmacogenomic markers. For instance, *DCT *is also a marker for melanoma and is melanocyte specific [65,67]. The melanoma cell lines in this case were all found in the sensitive group and this may serve as a confounding variable. Validation of our results in non-cell line data and in other tumor types are therefore necessary prior to clinical translation.

Working through the p53/β-catenin axis and microtubule interactions, our identified SNP variants can alter their respective gene products and in turn modulate the cellular response to paclitaxel causing the observed heterogeneity in both cellular and clinical responses.

### Requirement for an integrative approach in pharmacogenomics

Traditionally, the discovery of markers associated with drug response or prognosis of complex disease risk has depended upon investigating changes in the gene expression, in particular mRNA expression levels [72,73]. However, if this was applied here, only five of our genes would have been identified. Thus, it is important to use both genomic and expression data sets to complement each other as genomic variants which ultimately alter gene functions other than expression may not be detected [74]. Furthermore, it has been suggested that the discovery of such markers should use an integrated functional genomics analysis where information from genomic (i.e., mutation, copy number variation, SNPs, DNA methylation), expression and functional experiments should be combined to find genetic marker profiles [73]. This analysis could also include haplotype analysis which can reveal markers that are more meaningfully linked with traits, as done here, but also include analysis such as proteomics, epistatic changes and reverse-phase protein arrays (RPPA) [72,73,75].

A similar type of integrated analysis has been performed for paclitaxel by Park *et al*, where RPPA profiling along with mutation analysis and siRNA screening analysis was used to find and assess markers associated with paclitaxel sensitivity [26]. However, our results differed from those by Park *et al*, in part due to the different approaches used to identify markers associated with paclitaxel sensitivity. Our study focused on investigating a genetic association through categorization (sensitive or resistant) while Park *et al*, correlated sensitivity with protein expression level. As seen in our mRNA expression analysis, not all identified SNPs belonging to protein-coding genes showed changes in their respective gene's mRNA expression and this can hold true for protein expression as well. Also, although not investigated here, it is likely that other genes, not identified in our GWAS, show significant differences in expression levels between sensitive and resistant cell lines, despite not having any SNPs associated with drug response. As discussed previously, one possible mechanism for this could be through SNPs in other genes or intergenic SNPs altering the expression of genes on the same or different chromosomes and reinforces the need to complement expression data with genomic analysis [30]. Hence more integrative analysis approaches, integrating data from genetic (SNPs and copy number variations), mRNA expression, protein expression studies should be utilized to better understand the role of genetic variants in drug response.

## Conclusions

Our study has taken advantage of genotypic, expression and drug response data available for the NCI60 panel. Using this data, we have identified a number of genetic variants belonging to protein-coding genes that are associated with the cellular response to paclitaxel. Many of these variants may not have been identified solely on expression data emphasizing the importance of complementing expression data with genomic data. Through a series of bioinformatic and literature investigations, we have been able to infer how these SNP variants may alter paclitaxel response. Our identified haplotypes can potentially serve as better predictors of paclitaxel response as they have a stronger association with paclitaxel response than their individual SNP markers. Despite the potential limitations to using a cell line system, such as its accuracy in representing an *in vivo *system, our use of cell lines has allowed us to use well characterized and high throughput data, enabled us to focus more on variants directly affecting drug response instead of those affecting paclitaxel pharmacokinetics, while reducing the effect of confounding or non-genetic factors present in clinical studies. The genetic variants may play a significant role in the cellular response to paclitaxel, and represent potential biomarkers for predicting paclitaxel response.

## Authors' contributions

LE helped to design the study, carried out the GWAS, bioinformatic analysis, haplotype analysis, pathway-based and literature analyses, obtained the expression data and conducted expression analysis and drafted the manuscript. II helped to design the study, helped to conduct the GWAS and expression analyses, assisted in the statistical analysis of the expression analysis, helped to critically revise the manuscript and provided general support to LE. HJ helped to design the study, collected the genetic and drug response data. SS helped to design the study and helped to critically revise the manuscript. MM assisted in the statistical analysis of the haplotype analysis. KIP helped to design the study and helped to critically revise the manuscript. HO conceived the study, participated in the design and coordination of the study, assisted in the pathway-based and literature analyses and helped critically revise the manuscript. All authors have read and approved the final version of the manuscript.

## Pre-publication history

The pre-publication history for this paper can be accessed here:

http://www.biomedcentral.com/1755-8794/4/18/prepub

## Supplementary Material

Additional file 1 **Case-control design for sensitive and resistant cell lines**. This table lists the cell lines which were classified to be sensitive from our kernel estimation on the left and those classified as resistant on the right. A normalized value of 1.2 was used as the cut-off in designating sensitive or resistant. (Log Value = logarithm of GI50 concentration (M), Normalized value = z-score of log value in normal distribution, SNP Data = shows whether the SNP-genotype was available, mRNA data = indicates whether mRNA expression data was available, Y = Available, N = Not Available). Click here for file
