# Supplementary material for: Bioinformatic analyses identifies novel protein-coding pharmacogenomic markers associated with paclitaxel sensitivity in NCI60 cancer cell lines
Source: BMC Med Genomics. 2011 Feb 11;4:18. doi: 10.1186/1755-8794-4-18 (PMC3050680; doi:10.1186/1755-8794-4-18)
Supplement: Additional file 1 — Case-control design for sensitive and resistant cell lines. This table lists the cell lines which were classified to be sensitive from our kernel estimation on the left and those classified as resistant on the right. A normalized value of 1.2 was used as the cut-off in designating sensitive or resistant. (Log Value = logarithm of GI50 concentration (M), Normalized value = z-score of log value in normal distribution, SNP Data = shows whether the SNP-genotype was available, mRNA data = indicates whether mRNA expression data was available, Y = Available, N = Not Available). [file 1755-8794-4-18-S1.DOC]

| Sensitive Cell Lines | | | | | | Resistant Cell Lines | | | | | |
| --- | --- | --- | --- | --- | --- | --- | --- | --- | --- | --- | --- |
| Cell Line Type | Cell Line Name | Log Value | Normalized Value | SNP Data | mRNA data | Cell Line Type | Cell Line Name | Log Value | Normalized Value | SNP Data | mRNA data |
| Breast | MDAN | -8.513 | -1.67261 | N | N | NSCL | EKVX | -6.624 | 1.49829 | Y | Y |
| Breast | MDAMB435 | -8.406 | -1.493 | Y | Y | NSCL | HOP92 | -6.504 | 1.69972 | Y | Y |
| CNS | SNB75 | -8.321 | -1.35032 | Y | Y | Renal | CAKI1 | -6.399 | 1.87597 | Y | Y |
| Colon | HT29 | -8.27 | -1.26471 | Y | Y | Renal | UO31 | -6.335 | 1.9834 | Y | Y |
| Leukemia | RPMI8226 | -8.24 | -1.21435 | Y | Y | Colon | HCT15 | -6.32 | 2.00858 | Y | Y |
| Breast | HS578T | -8.212 | -1.16735 | Y | Y | NSCL | LXFL529 | -6.277 | 2.08076 | N | N |
| Colon | HCT116 | -8.199 | -1.14553 | Y | Y | Ovarian | OVCAR4 | -6.23 | 2.15966 | Y | Y |
| Leukemia | HL60(TB) | -8.124 | -1.01963 | Y | Y | Renal | ACHN | -6.179 | 2.24527 | Y | Y |
| Colon | KM12 | -8.055 | -0.90381 | Y | Y | Breast | NCI/ADRR | -6.05 | 2.46181 | Y | Y |
| NSCL | NCIH522 | -8.043 | -0.88367 | Y | N |  |  |  |  |  |  |
| Leukemia | K562 | -8.042 | -0.88199 | Y | Y |  |  |  |  |  |  |
| Breast | MCF7 | -8.041 | -0.88031 | Y | Y |  |  |  |  |  |  |
| Melanoma | LOXIMVI | -7.997 | -0.80645 | Y | Y |  |  |  |  |  |  |
| CNS | SF539 | -7.991 | -0.79638 | Y | Y |  |  |  |  |  |  |
| Colon | COLO205 | -7.958 | -0.74098 | Y | Y |  |  |  |  |  |  |
| NSCL | NCIH460 | -7.91 | -0.66041 | Y | Y |  |  |  |  |  |  |
| Leukemia | CCRFCEM | -7.901 | -0.6453 | Y | Y |  |  |  |  |  |  |
| Melanoma | SKMEL5 | -7.872 | -0.59662 | Y | Y |  |  |  |  |  |  |
| Ovarian | IGROV1 | -7.87 | -0.59327 | Y | Y |  |  |  |  |  |  |
| Prostate | TSUPRI | -7.855 | -0.56809 | N | N |  |  |  |  |  |  |
| Colon | SW620 | -7.833 | -0.53116 | Y | Y |  |  |  |  |  |  |
| Colon | HCC2998 | -7.832 | -0.52948 | Y | Y |  |  |  |  |  |  |
| Leukemia | SR | -7.826 | -0.51941 | Y | Y |  |  |  |  |  |  |
| Ovarian | OVCAR3 | -7.82 | -0.50933 | Y | Y |  |  |  |  |  |  |
| Ovarian | OVCAR8 | -7.802 | -0.47912 | Y | Y |  |  |  |  |  |  |
| CNS | U251 | -7.801 | -0.47744 | Y | Y |  |  |  |  |  |  |
| Leukemia | MOLT4 | -7.755 | -0.40023 | Y | Y |  |  |  |  |  |  |
| Prostate | PC3 | -7.754 | -0.39855 | Y | N |  |  |  |  |  |  |
| Renal | SN12C | -7.728 | -0.3549 | Y | Y |  |  |  |  |  |  |
| Prostate | DU145 | -7.666 | -0.25083 | Y | Y |  |  |  |  |  |  |
| Melanoma | M14 | -7.658 | -0.2374 | Y | Y |  |  |  |  |  |  |
| NSCL | NCIH23 | -7.655 | -0.23236 | Y | Y |  |  |  |  |  |  |
| CNS | SF268 | -7.625 | -0.18201 | Y | Y |  |  |  |  |  |  |
| NSCL | A549/ATC | -7.611 | -0.15851 | Y | Y |  |  |  |  |  |  |
| Ovarian | SKOV3 | -7.593 | -0.12829 | Y | Y |  |  |  |  |  |  |
| Melanoma | UACC62 | -7.592 | -0.12661 | Y | Y |  |  |  |  |  |  |
| NSCL | NCIH322M | -7.582 | -0.10983 | Y | Y |  |  |  |  |  |  |
| Melanoma | SKMEL28 | -7.575 | -0.09807 | Y | Y |  |  |  |  |  |  |
| Melanoma | MALME3M | -7.535 | -0.03093 | Y | Y |  |  |  |  |  |  |
| Breast | BT549 | -7.523 | -0.01079 | Y | N |  |  |  |  |  |  |
| Renal | 7860 | -7.462 | 0.09161 | Y | Y |  |  |  |  |  |  |
| CNS | SNB19 | -7.429 | 0.147 | Y | Y |  |  |  |  |  |  |
| NSCL | HOP62 | -7.41 | 0.1789 | Y | Y |  |  |  |  |  |  |
| Ovarian | OVCAR5 | -7.392 | 0.20911 | Y | Y |  |  |  |  |  |  |
| NSCL | NCIH226 | -7.375 | 0.23765 | Y | Y |  |  |  |  |  |  |
| Renal | RXF393 | -7.336 | 0.30311 | N | N |  |  |  |  |  |  |
| Melanoma | SKMEL2 | -7.295 | 0.37194 | Y | Y |  |  |  |  |  |  |
| Melanoma | UACC257 | -7.29 | 0.38033 | Y | Y |  |  |  |  |  |  |
| Breast | T47D | -7.288 | 0.38369 | Y | N |  |  |  |  |  |  |
| Breast | MDAMB231 | -7.261 | 0.42901 | Y | Y |  |  |  |  |  |  |
| CNS | SF295 | -7.16 | 0.59855 | Y | Y |  |  |  |  |  |  |
| Renal | TK10 | -6.919 | 1.00309 | Y | Y |  |  |  |  |  |  |
| Renal | A498 | -6.903 | 1.02995 | Y | Y |  |  |  |  |  |  |
